# Multitarget Drug Design, Molecular Docking and PLIF Studies of Novel Tacrine−Coumarin Hybrids for the Treatment of Alzheimer’s Disease

**Published:** 2018

**Authors:** Masood Fereidoonnezhad, Azar Mostoufi, Maryam Eskandari, Samaneh Zali, Fariba Aliyan

**Affiliations:** *Department of Medicinal Chemistry, School of Pharmacy, Ahvaz Jundishapur University of Medical Sciences, Ahvaz, Iran.*

**Keywords:** Multi-target-directed ligand, Drug-likeness, Molecular docking, PLIF studies, Alzheimer’s disease

## Abstract

Alzheimer’s disease (AD) as a complicated and progressive neurodegenerative disorder is the most common form of dementia and memory loss. On account of the multifactorial etiology of AD, the multi-target-directed ligand (MTDL) approach is a promising method in searching new drug candidates for this disease. Here, in this paper more than 500 tacrine-coumarin hybrids have been designed and drug-likeness, molecular docking and descriptor analysis of them were performed to find out a drug candidate with less toxicity and better binding affinity than tacrine. The docking analysis was carried out using human acetylcholineesterase (1ACJ), human butyrylcholineesterase (4BDS) and β-secretase (BACE1) (1W51) enzymes using AutoDock 4.2 and Vina. The promising results were obtained on the types of interactions. Based on docking on three targets and PLIF studies, the compounds that have better results were introduced as good candidates for synthesis. The validity of docking protocols was verified using a set of known active ligands and decoys on these targets.

## Introduction

Alzheimer disease (AD) is a complex neurodegenerative process occurring in the central nervous system (CNS), characterized by deposits of improper proteins namely β-amyloid (Aβ) and neurofibrillary tangles, inflammatory intermediates, reactive oxygen species (ROS), loss of synapses, and death of cells such as cholinergic neurons (1, 2).

The most therapeutic approach for the treatment of AD are drugs that aim to inhibit enzymes acetylcholineesterase (AChE) and butyrylcholineesterase (BuChE), thereby increasing acetylcholine concentration in cholinergic synaptic clefts (3, 4). Another rational therapeutic approach for treating AD is lowering the concentration of Aβ peptide in the brain (5). This purpose can be attained by decreasing Aβ production through inhibition of β-secretase (BACE-1) (6, 7).

Tacrine is a potent inhibitor of both AChE and BuChE that suffers from therapy-limiting liver toxicity, which can be prevented with free radical scavengers (8). Thus, the development of tacrine based dimers and hybrids with improved pharmacological properties and decreased side effects has been the focus of a great deal of research in recent years (8-10).

Recent studies have shown that coumarin has antioxidant effects and exhibits potent AChE, BuChE inhibition activity, therefore this compound being seen as potential drug in the treatment of AD (8). 

The multifunctional nature of AD provides the logical foundation for the development of an innovative drug design strategy centered on multi-target-directed-ligands (MTDLs). The multitarget approach has been proposed as particularly suitable to combat the heterogeneity of AD. In recent years, the MTDL concept has been exploited to design different ligands hitting different biological targets (11, 12). MTDLs can be produced by molecular hybridization (MH) (13). 

On the basis of our knowledge of the well-known structure of AChE, BuChE and BACE-1, we decided to connect the tacrine and coumarin fragments using hydroxyethylamine (HEA) as a linker (14, 15). This core has been applied successfully to a number of aspartyl proteases, such as beta-secretase 1.

In order to predict the biological affinity and activity of the small molecule drug candidates, molecular docking is mostly applied. Therefore, docking plays a great role in the rational drug design. Given the biological and pharmaceutical importance of molecular docking, significant efforts have been conducted towards improving the methods used to predict docking (16, 17). 

Here, in this study a library with more than 500 tacrine-coumarin analogues has been designed using MTDLs strategy. Drug-likeness, molecular docking, descriptor analysis, and protein-ligand interaction fingerprints (PLIFs) of them were performed to find out a drug candidate with better binding affinity and less toxicity than tacrine. Using a set of known active ligands and decoys, the validity of docking protocol was also determined.

## Experimental

*Preparation of the structures*

The three dimensional crystal structure of AChE (PDB ID: 1ACJ), BuChE (4BDS), and BACE-1 (1W51) were retrieved from protein data bank (18). Water and co-crystal ligand molecules were excluded from the structures and the PDBs were corrected in terms of missing atom types by modeller 9.12 (19). An *in-house* application (MODELFACE) was used for generation of python script and running modeller software. The enzymes were then converted to PDBQT by adding gasteiger partial charges using MGLTOOLS 1.5.6 (20).

*Designing of the ligands*

**Scheme 1:**
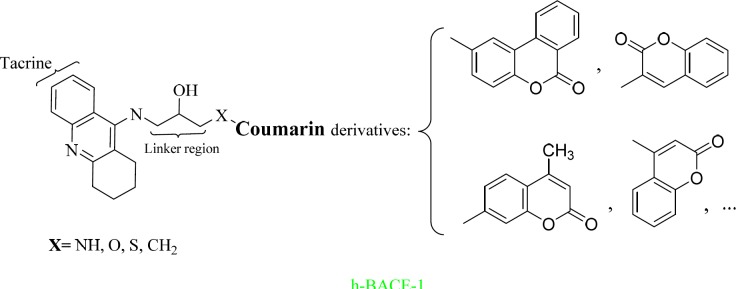
Designing of tacrine-coumarin hybrids using MTDLs strategy

More than 500 ligands were designed based on Scheme 1 using MTDLs strategy. The tacrine fragment was selected for its inhibition of AChE and BuChE. The coumarin scaffold was chosen for its β-secretase 1 (BACE-1) inhibitory and antioxidant activities. Based on the literature survey, the hydroxyethylamine linker was selected to have BACE-1 inhibitory activities.

*Optimization of the ligands*

The two dimensional structures of the ligands were drawn using ChemBioDraw Ultra v.13 software (Cambridge Software). Then, the ligands were subjected to minimization procedures by means of an *in house *TCL script using Hyperchem (Version 8, Hypercube Inc., Gainesville, FL, USA). Each ligand was optimized using molecular mechanics method (MM^+^) followed by quantum based semiemprical method (AM1) utilizing HyperChem 8. The output structures were thereafter converted to PDBQT by means of MGLtools 1.5.6 for docking procedure.

*Drug-likeness analysis*

Drug-likeness rules are set of principles for the structural properties of compounds, used for fast calculation of drug-like properties of a molecule. They can be quite effective and efficient. Using DruLiTo (21), an open source virtual screening tool, as it was shown in Table 1, drug-likeness descriptors such as Molecular Weight (MW), logP, AlogP, H-Bond Acceptor (HBA), H-Bond Donor (HBD), Total Polar Surface Area (TPSA), Atom Molar Refractivity (AMR), number of Rotable Bond (nRB), number of Atom, number of Acidic group, Rotatable bond Count (RC), number of Rigid Bond (nRigidB), nAtomRing, and nHB for all of the ligands were calculated. DruLiTo calculations is based on the various drug likeness rules like Lipinski›s rule, Veber rule, Ghose filter, BBB rule, CMC-50 like, rule and Quantitative Estimate of Drug-likeness (QED). The compounds that pass the drug-likeness filter were subjected to docking studies.

**Table 1:** Drug-likeness descriptors of compounds 1-34, calculated by DruLiTo software

**No.**	**MW**	**logP**	**AlogP**	**HBA**	**HBD**	**TPSA**	**AMR**	**nRB**	**nAtom**	**RC**	**nRigidB**	**nAromRing**	**nHB**
1	439.99	2.684	0.175	6	0	47.89	142.83	6	35	6	34	4	6
2	470.93	2.336	0.872	6	0	81.03	139.92	7	34	5	31	3	6
3	490.95	2.346	-0.391	7	0	64.96	146.22	9	37	5	32	3	7
4	474.95	2.317	0.251	6	0	55.73	144.04	8	36	5	32	3	6
5	472.96	2.781	0.278	6	0	38.66	149.23	6	36	6	35	4	6
6	470.96	3.39	0.712	5	0	38.66	148.45	6	36	6	35	4	5
7	490.93	3.442	1.289	5	0	63.96	153.73	6	36	6	35	4	5
8	461.92	2.907	0.368	6	0	47.89	134.07	6	33	5	31	3	6
9	457.93	3.036	0.43	5	0	38.66	134.89	6	33	5	31	3	5
10	477.9	3.088	1.006	5	0	63.96	140.17	6	33	5	31	3	5
11	487.93	2.419	0.139	7	0	55.73	139.34	7	35	5	32	3	7
12	470.96	3.39	0.712	5	0	38.66	148.45	6	36	6	35	4	5
13	447.98	1.135	-0.69	8	0	64.96	134.84	8	35	5	31	3	8
14	463.95	1.53	-0.132	7	0	81.03	141.29	8	35	5	31	3	7
15	389.99	2.124	-0.635	6	0	38.66	126.32	6	31	5	29	3	6
16	387.99	2.093	-0.201	5	0	38.66	125.54	6	31	5	29	3	5
17	391.99	1.964	-0.369	6	0	47.89	124.64	6	31	5	29	3	6
18	407.96	2.356	0.376	5	0	63.96	130.81	6	31	5	29	3	5
19	387.99	2.093	-0.308	5	0	38.66	125.46	6	31	5	29	3	5
20	407.96	2.145	0.269	5	0	63.96	130.74	6	31	5	29	3	5
21	455.98	1.98	-0.76	7	0	64.96	141.5	9	36	5	31	3	7
22	443.98	1.819	0.32	7	0	64.96	137.52	8	35	5	31	3	7
23	439.99	1.951	-0.118	6	0	55.73	139.32	8	35	5	31	3	6
24	441.99	1.288	-0.224	7	0	55.73	138.67	8	35	5	31	3	7
25	459.98	1.456	-0.625	8	0	74.19	139.88	9	36	5	31	3	8
26	455.98	1.588	-1.063	7	0	64.96	141.68	9	36	5	31	3	7
27	457.98	0.925	-1.169	8	0	64.96	141.03	9	36	5	31	3	8
28	469.98	1.691	-0.587	8	0	64.96	145.64	10	37	5	31	3	8
29	435.99	3.024	0.343	5	0	38.66	143.73	6	35	6	34	4	5
30	455.96	3.076	0.92	5	0	63.96	149.01	6	35	6	34	4	5
31	480.95	0.97	-0.865	8	0	55.73	140.71	8	36	5	32	3	8
32	442.93	2.722	0.744	5	0	63.96	135.53	6	32	5	30	3	5
33	424.96	1.85	-0.373	6	0	38.66	130.96	6	32	5	30	3	6
34	461.92	2.696	0.262	6	0	47.89	134	6	33	5	31	3	6

*Docking procedure*

The docking simulations were carried out using an *in house *batch script (DOCKFACE) for automatic running of AutoDock 4.2 and AutoDock Vina. In all experiments genetic algoritm search method was applied to determine the best pose of each ligand in the active site of the target enzymes. The genetic algorithm and grid box parameters for our three targets are listed in Table 2. Random orientations of the conformations were generated after translating the center of the ligand to a specified position within the receptor active site, and making a series of rotamers. This process was recursively repeated until the desired number of low-energy orientations was obtained. The docking was carried out on flexible ligands and rigid receptors.

**Table 2:** Genetic algorithm (GA) and grid box parameters

**Parameter Name**	**AChE**	**BuChE**	**BACE-1**	**GA** a ** Parameters**	**Value**
PDB ID	1ACJ	4BDS	1W51	Number of GA Runs	100
No. of points in x	40	50	50	Population Size	150
No. of points in y	40	50	50	Max. No. of evaluations	2500000
No. of points in z	40	50	50	Max. No. of generations	27000
Grid spacing	0.375	0.375	0.375		
Box X center	4.395	55.7	63		
Box Y center	69.901	46.5	-3.763		
Box Z center	65.807	81	75		

^a^Genetic algorithm.

*Analysis of docking results*

Having finished the docking process, the protein–ligand complex was analyzed in order to understand the type of interactions. Top ranked binding energies (kcal/mol) in AutoDock dlg output file were considered as response in each run. 

AutoDock Vina is a surrogate of AutoDock 4.2 and has a new knowledge-based, statistical scoring function instead of the semiempirical force field of AutoDock 4.2. Due to great prediction accuracy and speed over AutoDock 4.2, Vina results were selected as the best docking binding energies. Docking results were supported almost by high cluster populations. The best docking result in each case was considered to be the conformation with the lowest binding energy. Table 3 revealed the ligands with the best docking results in terms of its binding free energy to the receptors.

**Table 3:** Compounds with best docking binding scores

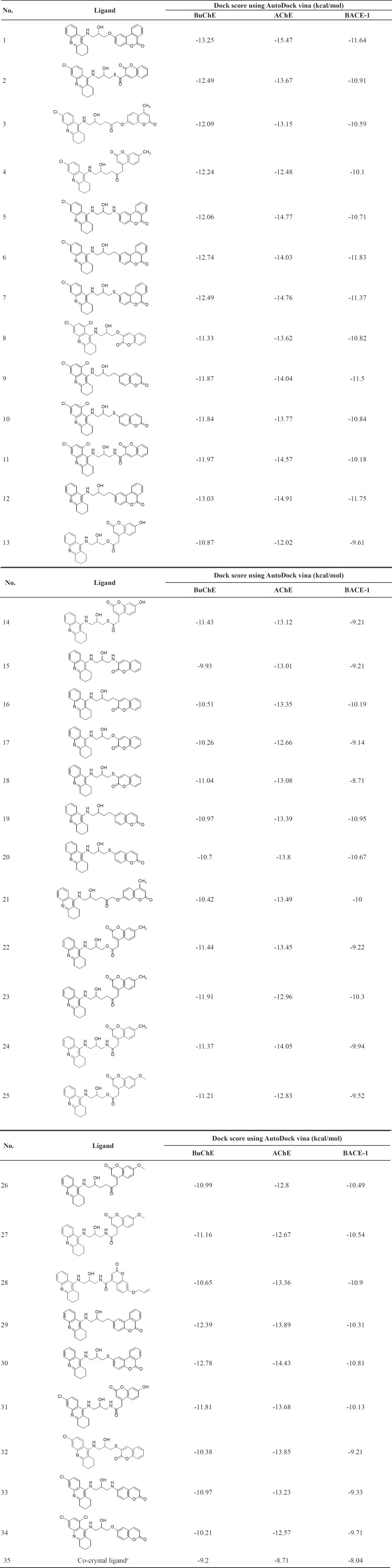

a In case of AChE and BuChE, the co-crystal ligand is Tacrine.

*Protein ligand interaction fingerprint (PLIF)*

In order to perform PLIF studies on docking results, by means of preAuposSOM application (22), the poses of docking were extracted from dlg files. The resulted PDBQTs and the receptor were converted to MOL2 be means of a batch script using Open Babel 2.3.1. The resulted mol2 files were subjected to AuposSOM 2.1 web server (23). Two training phases with 1000 iterations were set in the self-organizing map settings of AuposSOM conf files. Other parameters of the software were remained as default. The output files were subjected to Dendroscope 3.2.10 (24, 25) for visualization of the results. Dendroscope is a phylogenetic relationship software that is able to visualize rooted phylogenetic trees and networks efficiently.

## Results and Discussion

**Figure 1:**
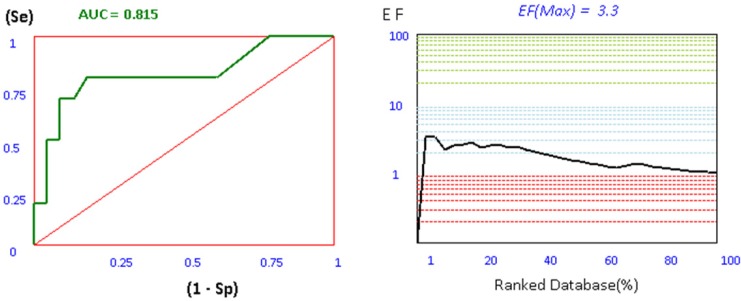
ROC and EF diagrams for AChE receptor

For investigation the validity of our docking process, a set of 106 AChE inhibitors, 161 BACE-1 inhibitors, and 42 BuChE inhibitors were retrieved from ChEMBL database as SMILES format (26-28). Iterative runs of Open Babel 2.3.2 through a shell script provided the primary 3D generation of the structures as MOL2 format. In order to use this metric in a virtual screening (VLS) study, the ligands must be first categorized in to two subsets of actives and decoys based on their experimental activities. Afterwards, this ligands and decoys were docked by our set up docking procedure. The application of ROC in computational medicinal chemistry was widely used as a useful metric in order to evaluate the validity of docking scores in VLS studies. ROC plots are subsequently being obtained by plotting (Se) versus (1-Sp) for all docking scores. The area under the curve for ROC plots is calculated by trapezoidal integration method as implemented in our application. The more ROC_AUC_ value means that the docking protocol is more able to discriminate between active ligands and decoys. Another tool to evaluate the efficiency of docking protocol in VLS studies is enrichment factor. Compared to ROC plot, EF_max_ factor is highly dependent on the number of actives in a data set. Since ROC values do not depend on the number of actives and decoys, they are more valuable in making decisions about the validity of the methods than EF_max_ analysis. The plots and results of ROC and EF_max_ provided for AChE are depicted in Figure 1.

**Figure 2:**
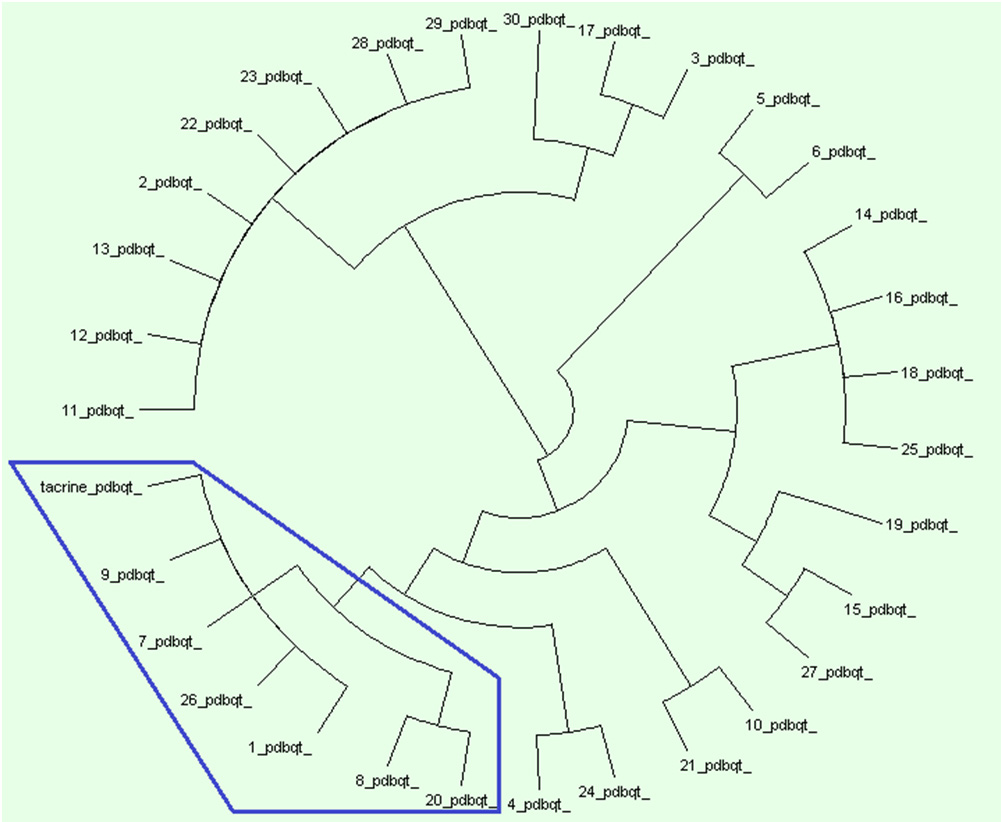
AuposSOM results for poses of docking. AuposSOM 2.1 web server results analysed by Dendroscope

Protein ligand interaction fingerprint (PLIF) as another reliable analysis technique is used mainly in computational chemistry studies. This method makes it possible to study the effect of different starting states of the structures on generated poses as well as their corresponding vector of contacts towards receptor during docking procedure (29). As it was previously described, all generated poses of ligands and the tacrine were subjected to AuposSOM 2.1 to calculate their contact vectors within the receptor binding cavity. In this method, the contacts between the ligands and the receptor include hydrophobic, hydrogen bonding, and coulombic interactions. The resulted vectors of contacts are subsequently analyzed using self-organizing map as implemented in AuposSOM software. The output of self-organizing map is a classification pattern for ligands. As it was shown in Figure 2, tacrine with ligand numbers 1, 7, 9, 26, 8, and 20 are in a same subgroup. Since compounds in the same subgroup may show a similar behavior, so these compounds can be good candidates for synthesis. PLIF is another interpretation on docking results.

**Figure 3:**
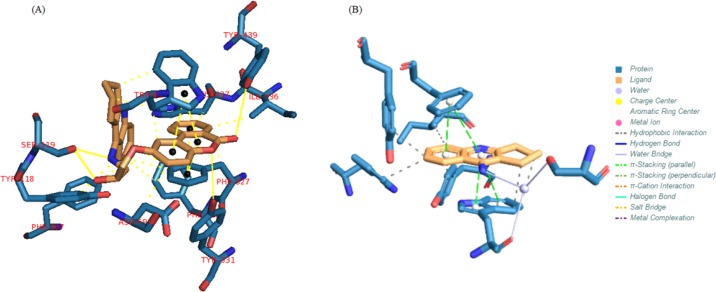
(A) Interactions of compound 1 with the residues in the binding site of AChE receptor (1ACJ). (B) Tacrine interactions with 1ACJ

The results for each ligand were compared to its corresponding co-crystal ligand. Binding interactions between docked potent agents and the targets was analyzed using AutoDock tools program (ADT, Version 1.5.6) and PLIP (fully automated protein–ligand interaction profiler) (30). As it was shown in Figure 3a, three types of interactions such as hydrogen bond, π-Stacking, and hydrophobic exist between compound 1 and AChE receptor. A hydrogen bond interaction exists between hydroxyl of HEA and NH of tacrine moiety in this compound with Ser119 and there is also a hydrogen bond existing between carbonyl group of coumarin moiety with TYR439 and its oxygen with TYR331 in the receptor. Due to the great interaction between compound 1 and AChE receptor, the other interaction is summarized in Figure 3a, meanwhile, tacrine have π-Stacking interactions with TRP84 and PHE330 as well as some hydrophobic interactions (Figure 3b).

**Figure 4:**
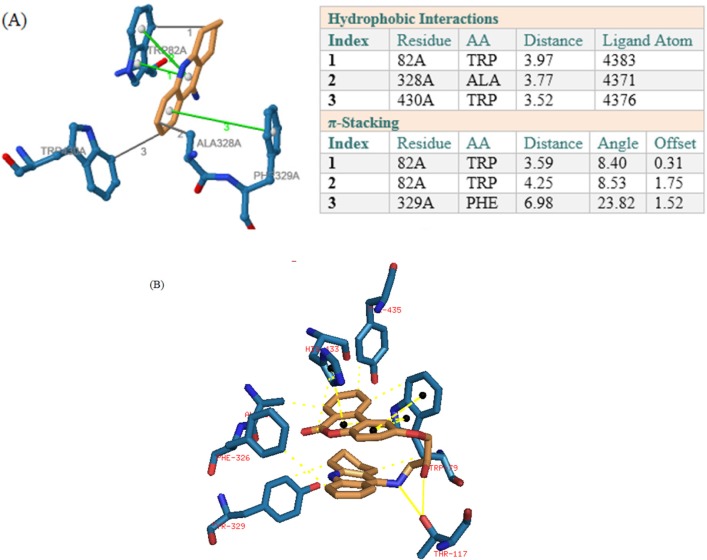
(A) Interactions of tacrine with the residues in the binding site of BuChE receptor (4BDS). (B) Compound 1 interactions with 4BDS

In BuChE receptor, tacrine interacts via π-Stacking bonds with TRP89 and PHE329 and some hydrophobic interactions which were shown in Figure 4a. Compound 1 interacts via hydrogen bonds through its hydroxyl of HEA and NH of tacrine moiety with THR117. As it was depicted in Figure 4b, there are also some π-Stacking interactions with TRP79 and HIS433 as well as some hydrophobic interactions.

**Figure 5:**
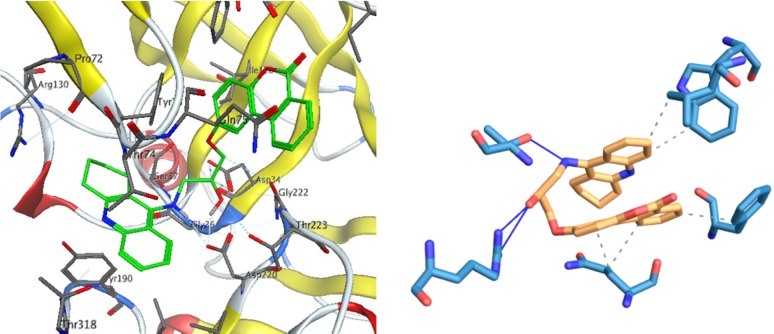
Interactions of compound 1 with the residues in the binding site of BACE-1 receptor (1W51)

In BACE1 binding mode, hydroxyl group of compound 1 interact via two hydrogen bonds with ARG227 and also a hydrogen bond exists between NH of tacrine moiety with THR224. The hydrophobic interactions are shown in Figure 5. 

## Conclusion

Here, we applied MTDL approach as a promising method in searching new drug candidates for alzheimer’s disease. More than 500 tacrine-coumarin hybrids have been designed using MTDL strategy. The molecular docking analyses as well as protein-ligand interaction fingerprints studies showed that 34 ligands are effective in their docking binding energies and high binding natures to AChE, BuChE and BACE-1 receptors. Thus, these analogues are good candidates for synthesis and to develop an effective multifunctional drugs for the treatment of alzheimer’s disease should be considered for further evaluation using *in-vitro* and *in-vivo* studies.
